# The challenge of social networking in the field of environment and health

**DOI:** 10.1186/1476-069X-11-S1-S15

**Published:** 2012-06-28

**Authors:** Peter van den Hazel, Hans Keune, Scott Randall, Aileen Yang, David Ludlow, Alena Bartonova

**Affiliations:** 1 Public Health Services Gelderland-Midden, Arnhem, The Netherlands; 2 Research Institute for Nature and Forest (INBO), Brussels; Centre of Expertise for Environment and Health, Faculty of Political and Social Sciences, University of Antwerp; naXys, Namur Center for Complex Systems, University of Namur, Belgium; 3 NILU - Norwegian Institute for Air Research, Kjeller, Norway; 4 Centre for Sustainable Planning and Environments, University of the West of England, Bristol, UK

## Abstract

**Background:**

The fields of environment and health are both interdisciplinary and trans-disciplinary, and until recently had little engagement in social networking designed to cross disciplinary boundaries. The EU FP6 project HENVINET aimed to establish integrated social network and networking facilities for multiple stakeholders in environment and health. The underlying assumption is that increased social networking across disciplines and sectors will enhance the quality of both problem knowledge and problem solving, by facilitating interactions. Inter- and trans-disciplinary networks are considered useful for this purpose. This does not mean that such networks are easily organized, as openness to such cooperation and exchange is often difficult to ascertain.

**Methods:**

Different methods may enhance network building. Using a mixed method approach, a diversity of actions were used in order to investigate the main research question: which kind of social networking activities and structures can best support the objective of enhanced inter- and trans-disciplinary cooperation and exchange in the fields of environment and health. HENVINET applied interviews, a role playing session, a personal response system, a stakeholder workshop and a social networking portal as part of the process of building an interdisciplinary and trans-disciplinary network.

**Results:**

The interviews provided support for the specification of requirements for an interdisciplinary and trans-disciplinary network. The role playing session, the personal response system and the stakeholder workshop were assessed as useful tools in forming such network, by increasing the awareness by different disciplines of other’s positions. The social networking portal was particularly useful in delivering knowledge, but the role of the scientist in social networking is not yet clear.

**Conclusions:**

The main challenge in the field of environment and health is not so much a lack of scientific problem knowledge, but rather the ability to effectively communicate, share and use available knowledge for policy making. Structured social network facilities can be useful by policy makers to engage with the research community. It is beneficial for scientists to be able to integrate the perspective of policy makers in the research agenda, and to assist in co-production of policy-relevant information. A diversity of methods need to be applied for network building: according to the fit-for-purpose-principle. It is useful to know which combination of methods and in which time frame produces the best results.

Networking projects such as HENVINET are created not only for the benefit of the network itself, but also because the applying of the different methods is a learning tool for future network building. Finally, it is clear that the importance of specialized professionals in enabling effective communication between different groups should not be underestimated.

## Background

The lack of a structured and integrated international network encompassing the entire field of stakeholders in the domains of environment and health is apparent. Furthermore, there is no common platform for interaction between policy makers, scientists and other societal representatives. The European Commission requested the organisation and structuring of networking activities between a diversity of actors in the field of environment and health in the HENVINET (Health and Environment Network) project, an FP6 project funded by DG Research. The core concept is that by facilitating interactions between professionals across disciplines and specialisations, an interdisciplinary and trans-disciplinary social network could lead to an enhanced problem solving potential. Inter- and trans-disciplinary networks are frequently considered as a useful means of enhancing communication and cooperation between different actors in order to raise the problem solving potential of both science and policy makers. However, such networks are not easily organized, as openness to such cooperation and exchange is often lacking [1].

The aim of this study is to analyse which network building actions are most efficient. Questions arising in this respect include how could the diverse actors in environment and health learn from each other, listen to each other, and find ways to cooperate and exchange knowledge? HENVINET investigated the complex relationship between disciplines, and how the actors could open up to networking in order to enhance the possibilities for solving problems, exchange of knowledge on good practice and understanding each other’s role. The main question is what kind of social networking activities and structures can be most supportive in delivering enhanced inter- and trans-disciplinary cooperation and exchange?

The main objective of HENVINET was to establish an inter- and trans-disciplinary network of professionals active in different disciplines and at different levels including local, regional, national or international. Sub-objectives were related to 1) the expectations of a network, 2) the most relevant policies to be addressed by a network, 3) the structure, organisation and provision of knowledge to be used by a trans-disciplinary network and, 4) the practical issues related to dissemination and outreach to potential stakeholders. The first, third and fourth sub-objectives are addressed in this paper, formulated into the following questions:

1) What gap can an inter- and trans-disciplinary network of different professionals fill in integrating the domains of environment and health? (*What is the need?*)

2) How can the production and exchange of knowledge be organized and improved by means of an inter- and trans-disciplinary network? (*What are the organizational aspects of a network?*)

3) How can an inter- or trans-disciplinary network be positioned in such a way that it serves the needs of a substantial portion of the experts, including policy makers and other actors? (*How can it reach out?*).

4) What are the results or outcomes of the different methods of creating an inter- or trans-disciplinary network, and how can lessons be drawn for future network building activities? (*What did we learn from different applied methods?*)

In this study experimentation with a diversity of actions took place in order to investigate the main research question: what kind of social networking activities, actions and structures can be most supportive in reaching the objective of enhanced inter- and trans-disciplinary cooperation and exchange in the fields of environment and health.

As with most EU-funded projects the starting point for network building in HENVINET was focused on traditional communication, specifically dissemination of the work undertaken in the project; traditional in the sense of a one-way communication from experts to target audience. At the start of the project the aims and research methods of the consortium were defined and the publications to be prepared for the EU for dissemination purposes identified. In addition, information on the project was promoted via the HENVINET project website (http://henvinet.nilu.no), which was progressively developed. Newsletters, website, leaflets were some of the traditional tools used for project dissemination. The actual use of this information material by the broad range of stakeholders was largely unknown. The website statistics did not provide any definitive information on the usage of the scientific information provided. The experience of the authors from previous EU projects was that the outputs of research projects often fail to survive for long after the end of the project. There are no mechanisms in place in EU research projects to maintain project collaborators in a network, either formally or informally, after the end of EU contract funding.

## Methods

HENVINET promoted a number of activities in order to facilitate experiment, and to meet the stated goals to develop understanding between diverse actors in order to improve the process of trans-disciplinary communication via networking.

### Stakeholder Interviews

HENVINET conducted interviews with a diversity of actors to identify the needs of policy makers and other stakeholders for information from the scientific community. In particular, interviews were conducted to obtain inputs from a diversity of stakeholders concerned with the construction of an inter- and trans-disciplinary network in the fields of environment and health. The protocol for the interviews is included as a supplementary file (Additional file 1)

### Respondees

Candidates for interviews were proposed by the HENVINET consortium (scientists, policy experts, and some advocacy stakeholder groups), and a range of experts from both the environment or (public) health fields were engaged. The HENVINET partners prepared a list of policy experts located at regional, national and Inter Governmental Organisation levels, which all had some connection with environment and health. The potential interviewees were contacted by telephone by members of the consortium, an appointment was made for a personal interview, and in a few cases where the distance between the partner and the interviewee was great e.g. Argentina, a telephone interview was held. All interviewees received the questions by email in advance, and the answers to the questions were compiled in English.

### Networking portal

Evidence from other EU projects indicated that networks, consisting of a diversity of disciplines in environment and health, are difficult to maintain [1,2], and accordingly a novel approach was sought. Different actions to secure actor involvement, both within and outside the consortium, were considered, ranging from classical approaches to knowledge exchange using reports to establish a social network. HENVINET examined the option of establishing a virtual network of actors from different disciplines, and decided to pursue this option by building an internet based network portal, to provide a structural tool for inter- and trans-disciplinary networking. A virtual network facilitates communication amongst a large group of different actors, and can be viewed as a means of providing a dynamic for social networking. There is little known about the long term effects of virtual social networks, and most existing networks are used for building professional contacts (such as Linkedin) or to exchange information on any given topic. Social interdisciplinary networks with the aim of supporting the policy process were not identified in the literature.

### A role-play session

Role-Playing Games (RPG) are used as Information and Communication Technology (ICT) tools that aim to provide support for educational activities, and for analysis and support for negotiation processes [3]. RPG aim at providing participants with improved knowledge of a given case or situation, reproducing part of the complexity of any issue in order to assist scientific and/or stakeholders understanding. From analysis to support, RPG are involved either collectively or individually in various negotiation processes. The design of RPG is not standardized, therefore they should be used as a tool based on an empirical approach, and should address awareness of behavioural patterns through the specification of roles and rules, as well as learning about the behaviour and viewpoints of players [3]. Barreteau states: “RPG aim at simulating complex systems such as those that are at stake in negotiation processes. These simulations are based on the background assumption that it is useful to control part of this complexity in order to (i) better grasp the consequences of the controlled part and (ii) make the other part react to the situation proposed by the controlled part” [3]. To the best of our knowledge, the network building capacity of role-play has not been reported in the literature.

### Voting session

A Personal Response System (PRS) is a form of technology that permits an audience to reply to questions or statements individually by selecting an answer on a hand-held wireless transmitter. The answers are collected by receivers connected to a computer. Computer software aggregates the responses, and the results are projected on a large screen using a standard beamer and software.

The PRS is very easy to use and offers a method of active engagement. Some research has found that it has a very significant effect on students’ performance in lectures, stimulating their interest and concentration [4] and creating greater engagement and broader participation [5]. Furthermore, it increases the audience’s enjoyment of lectures, and it has proved to be an excellent method of encouraging active learning. There is no data about the role of PRS in building trans-disciplinary networks, and this technique has not been used in the field of environment and health before.

### Stakeholder workshop

HENVINET conducted a workshop for stakeholders including policy professionals working on the theme of climate change in cities (for an extensive report on this workshop see [6]). The European Commission (EC) White Paper “Adapting to climate change: Towards a European Framework for Action” [7], for example as a frame of reference, considers the necessary adaptation responses of the EU and the member states in defining a framework for action in response to climate change, including human health. A workshop is a common forum to bring actors from different fields together.

## Results

### Stakeholder interviews

The geographic origin of interviewees was 74% from Europe and 26% from outside Europe. The period of the interviews was between July 2007 and April 2008, and a total number of 23 interviews were performed.

At the commencement of the interviews the purpose of building a network within the HENVINET project was explained, and subsequent questions related to the establishment of this network included: ‘Do you have comments, suggestions, and concerns about the HENVINET network? What do you expect from HENVINET?’ To these open questions four different key word response options were provided which related to a role or expectation from the network including: information, cooperation, dissemination and policy.

The key word *information* was mentioned by most of the interviewees (n=23). In addition, the interviewees had more suggestions for what kind of information is needed or how this should be presented. These included information as review of research, stimulation of empirical multidisciplinary research, collection of data (showing associations between environmental factors and human health), provision of access to data- bases, access to information at the local level, identification of knowledge gaps, and finally gathering information from other research fields.

Cooperation between a diversity of actors was mentioned by interviewees as an opportunity for a network to maximise the distribution of the available results. Interviewees also stressed that the European dimension provides added value with the respect to distribution, such that results could be disseminated internationally. Interviewees emphasised the need for extension of the network to include other, new stakeholders, if it intends to maintain its growth in the future. As stated by one interviewee: ‘the challenge is not the lack of information and research results, but we are not able to make knowledge available for decision making.’

The role of the network in respect of dissemination was identified by interviewees as a role interacting with different actors, but more specifically it is viewed as an opportunity to secure gain closer collaboration between policy makers and researchers. This role is identified as critical in the responses of the interviewees, confirming the need for an integrated approach, the formation of a network, and we ensuring the trans-disciplinary approach of the portal.

A specific role for the portal was identified in the translation of scientific information to vulnerable groups. Interviewees suggested that this could be done in a practical way with leaflets targeted at schools, hospitals or public authorities. The interviewees were clear about the need for a network in the field of environment and health, and although a challenge was identified in remaining fully up-to-date in the dynamic field of health and environment, this might be assisted by involving as many experts as possible in order to keep the information up to date. This recommendation links to the role of an intermediary, and the different roles Jeffrey [8] identifies for cross-disciplinary research. The intermediary is accepted by all parties on grounds of the mediator's integrity and good will. Jeffrey states that ‘the disciplinary groups need to believe that the intermediary is a credible and competent individual, and that he or she has the best interests of the project as a whole at heart” [8]. The intermediary is an effective communicator and experienced in operating intellectually in more than one disciplinary area.

The third research question dealt with the positioning of a network to fulfil the needs of a substantial portion of the experts. A first step was identified, by the interviewees, with regard to the policy field, namely the need to make a distinction between different groups of decision makers, and the view that the network should address the science-policy interface by providing clear and concrete policy recommendations, relevant to the different groups of decision-makers.

Indeed several distinct strands of opinion can be identified in the responses of interviewees. There is a group that want the network to heighten the awareness of policy makers with respect to the effects on health of environmental factors. This group suggests that we need products that can be used by politicians to improve access to information.

Another group wants the communication improved between researchers and decision makers. This includes an increased awareness of researchers about their own objectives, their own interests and that of policy makers. A suggestion was also made that the network should develop the concept of the scientist for global responsibility.

Another strand of responses identifies the need for experts who can act at the interface between research and policy to influence policy making. The misleading and prevalent model in which knowledge flows from the scientist that produces, to the policy maker that consumes is according to this group of interviewees, an incorrect interpretation. When policy makers do not respond to scientific inputs one might identify a communication problem, but equally policy makers may have their own strategic requirements supporting selective use of scientific information. Therefore, as indicated by several interviewees, the network has to consider the construction of a robust and enduring structure by early engagement with stakeholders.

Finally, most interviewees identified the difficulties in building a network between the scientific and policy community. The starting point is that simple policies do not exist, and one measure is never the solution, so a plan consisting of a series of measures is necessary, as many factors are involved in the management of any environmental - health effect relationship. In this respect a notable suggestion was that the research community has to play a role in the development of integrated health policies.

Based on the interviews with different stakeholders, most of them working in the policy making field, a concept emerged focused on the establishment of a social, virtual network as a platform for communication between different stakeholders. This was seen as a possible way to position an inter- or trans-disciplinary network linking experts in the field of environment and health. In conclusion, the interviewees confirmed the need for an inter- or trans-disciplinary network.

### Networking portal

HENVINET developed a social, virtual network portal for a trans-disciplinary group of individuals working in the health and environment domain at http://www.henvinet.eu. The aim was to develop a parallel communication between scientists and policy experts, and also between scientists themselves, and between policy experts. This does not, however, mean that the communication is only limited to the scientific and policy communities. Other actors were also invited to participate and become a part of this virtual network. Up-to-date knowledge on the selected themes of HENVINET was made available via the portal, and current social networking tools, comparable to Facebook or Linkedin, were installed. Individuals registered for the portal, created a personal profile, and indicated their interests in specific topics and their own speciality. Automated notifications of new information uploaded on the website were installed, and several thematic discussion groups were formed.

The fourth research question dealt with the outcome of the different methods applied and the lessons which could be defined. The portal is relatively new, but some initial results are already available.

The structure of the portal is comprehensive, including functionalities concerning making new contacts, viewing related events, and discussing trans-disciplinary topics of interest [9,10]. The framework of the portal was sufficiently broad in scope to address and assemble content for the various sub-themes of the health and environment field, for example the HENVINET project topics of: cancer, neurodevelopmental disorders, asthma and allergy, endocrine disrupting effects, climate change and health, and nanoparticles. Other example topics addressed included: noise pollution, bio-monitoring, children’s health, and transport induced air pollution.

Whilst the design and structure of the portal is robust, overall participation within the networking portal during the project period was low. The quality of the content within the various thematic groups and topics was considered satisfactory, but the amount and diversity of content available was less satisfactory. These results suggest that whilst a functioning platform was offered to enhance social networking great, a gap or some blocking mechanism existed which prevented the virtual network from being effectively established and becoming sustainable.

### A role-playing session

HENVINET conducted a role-play session at one of the project annual meetings (April 2009). The role-play format was inspired by experience with the development and use of role play previously developed within the field of environment and health [11,12]: whereby a balance needs to be found between respect for the complexity of environment and health issues which the role play aims to discover and discuss, and the reality that the role play should not be too difficult to perform by the participants in order to fulfil its social learning capacity. In order to make the role-play easier to perform but also sufficiently illustrative of the complexity of reality, the discussion agenda was narrowed to one simple question. At the same time the diversity of actors involved in the discussion aimed to create the potential for the discussion to mirror the complexity of environment and health. The aim was, so to speak, to conceal the complexity of the situation behind the different social perspectives on what could be viewed, at first sight, as a simple issue.

The participants had to play roles, in small groups of two to four persons, representing stakeholders from different organisations such as national authorities, scientific organisations (as consultants), industry, public health authorities and NGO’s. The topic of the role-play was a discussion on the meaning of a policy brief on the environment and health risks of a pollutant: the role-play discussion by a diversity of actors aimed to provide the authorities with advice on measures to be taken regarding the pollutant, based on the expert advice in the policy brief. The aim of the role-play was on the one hand to test how a stakeholder discussion on such a policy brief evolved, and on the other hand to introduce stakeholder involvement to the participating experts. It thus aimed to perform a learning experience in different respects.

At the beginning the participating scientists were sceptical about the usefulness of such a session. Two moderators introduced the topic and the structure of the RPG. The roles were distributed among the participants of the session. These roles were randomly distributed. The roles were allocated to five different groups: local government, local residents, industry, non-governmental organisations, and public health authorities. The diversity in roles aimed to ensure that the complexity of the issues under discussion would be highlighted by the different perspectives and stakeholders. The moderators provided role-information at the start of the session. Most participants could use their own experience and knowledge to fit their role. First a plenary exchange of views was provided by the different role groups. In two rounds the issue at stake was debated and in plenary sessions views were exchanged. After the role-play the outcomes we presented at a plenary session of HENVINET in order to inform experts not present at the role-play about its findings.

The participants were free to choose a view or opinion on the issue. Each role was represented by three to four persons. Each group was then requested to present two arguments in favour of their view. The next step was a plenary exchange of views. Already during the first round it was clear that the industry group was in opposition to most other role-groups, for example the NGO’s. The moderators on occasion stimulated the discussion in the role-group by feeding them with additional information to develop discussion. Two rounds of argument and discussion followed in which the other role-groups defined their position. Finally, a plenary discussion about advice to local government actors was scheduled. After this the role-play group discussion and evaluation took place. In this session the participants learnt from each other the lessons that emerged, and how each group supported its own arguments. The sub-groups easily adopted the stereotype role of the stakeholder they represented. Industry was defensive, NGO’s greatly opposing industry views, experts requesting more research, and local authorities waiting for a decision. In the evaluation it was stated that the views of different social perspectives were most valuable.

The scientists performing the role of the NGO discovered how simple it was to use their own scientific knowledge to attack the polluter, the industrial representative. While the national authority representatives found it hard not to allow their scientific knowledge to prevail over the other issues they had to address including economic and social issues. The public health authorities were easily manoeuvred into the position of defending the general public’s interest and health, although internally they had difficulties in agreeing the level of scientific proof. As a result they became less interesting partners for both the national authorities and the NGO’s. Finally, the industrial representatives became defensive and deployed all available arguments concerning lack of scientific certainty to avoid any responsibility or claims of harm done.

### Voting session

Using PRS, the participants at the HENVINET Final Event were asked to participate at an interactive voting session in order to review feedback concerning the HENVINET portal and to develop suggestions for further deployment and development. Areas addressed at this session involving 53 participants included: analysis of stakeholders; needs of the participating stakeholders; involvement of stakeholders in network activities; science-policy interface.

The participants were mainly represented by researchers (44%), providers of public information on Environment and Health (17%), risk assessors (15%) and those related to the policy field (15%).

The majority of voters considered the most important feature of the HENVINET portal to be the provision of scientifically sound information provided by experts in the user’s field of interest. Detailed issues such as user friendliness or the value of an automatic system for notifying new items on the portal appeared to be less important.

Questions arose about the most important and desirable factors in the development of policy advice, and 50% of the participants agreed that the traditional evidence based culture is in need of critical discussion and innovation. Only a small number of voters favoured the view that scientific information, as presented during the conference, should continue to be used by policy makers for decision making. The full results are reported in [13].

### Stakeholder Workshop

The HENVINET workshop on integrated urban management - climate change and health impacts addressed a prime goal identified by the White Paper [7] concerning: integration of climate change adaptation and health within policy frameworks at both local and EU levels.

The workshop deployed the backcasting approach as a form of expert analysis, building on the experience and expertise of a multidisciplinary group of experts in response to the complexity of many issues. This complexity is identified in the risks associated with climate change adaptation and mitigation measures proposed at the urban level, and the associated uncertainties regarding outcomes in respect of human health, quality-of-life, and economic vitality. The methods and reasoning for this approach are fully explained in Keune et al. [6].

Presentations were given on behalf of the cities of Bristol, Prague, Bologna, Ancona, Tilburg, and Frankfurt, and it is evident that the cities are using a wide range of integrated management strategies in response to a range of environmental topics based on the varying geographical and historical conditions of each city.

A first observation arising from the HENVINET workshop, but similar to that seen in other workshops, is that the participants from organisations outside the project consortium are already active in the topic. Most of these stakeholders are seeking additional knowledge, want to exchange ideas with colleagues to increase the quality of their own policy making, or want to confirm their proposed policies.

A common message resulting from the city presentations was the need for caution in adopting strategies from cities with different structures i.e. most strategies are customised to the specific region they were developed for, and it may be inappropriate to simply export these strategies to new areas with locally differentiated requirements.

The backcasting exercise was based on an agreed common target statement for the year 2030 – the statement stresses the importance of a healthy population and cooperation towards this goal. The numerous opportunities and barriers to the attainment of this goal were discussed, in which many of the issues included factors such as economics, communication, public engagement, policy specifics, and local alliances. A major recurring issue, much discussed when developing common targets, concerned the lack of knowledge regarding the connection between climate change and specific health effects. There is sufficient knowledge to realize some actions, but this could become a bottle-neck in the future when more concrete measures need to be implemented.

The workshop was appreciated by all participants, and can be seen as a valuable exercise for cities in sharing their experience in formulating integrated management approaches addressing climate change and health issues. It is hoped that a permanent expert group can evolve from these workshops to provide a bridge between science and policy for enhanced collaboration between health and environment.

## Discussion

With regard to network building, activities used in HENVINET may be identified as a form of action research. They were used as drivers to produce practice-relevant results in building a network consisting of a diversity of actors. The ambition was to enable scientists, policy makers and other actors to interact and co-operate by involving them in the various activities. Participation in these activities aimed to enhance the understanding of each other’s position in the process of policy making. Social scientists supported the process of network building. The activities were established by an interdisciplinary group of actors from HENVINET, including (social) scientists, medical doctors, veterinarians, statisticians, epidemiologists, public health professionals, policy makers and other professionals. These activities were undertaken to enhance awareness by the participants of each other’s role in the environment and health policy making process. The relevance of such a mixed methods approach has been described [14], elsewhere, and the context in which the network building was defined was interdisciplinary triangulation, where several disciplines are used to inform the research process [15].

Since the EU FP5 programme network activities [16] have been developed within Coordination Action projects, and thematic networks, as a new form of research project. However, all these networks have been scientifically oriented and had difficulties in engaging with policy related issues. Some networks produced reports on stakeholder analysis (e.g. AIRNET, NoMiracle, INTARESE) but all projects had difficulties in establishing stable connections to policy makers. No continuous network with a trans-disciplinary character was established.

One positive effect from these networks has been the establishment of more frequent contact between the scientific community and the multinational knowledge and data oriented organisations, most of them funded by the EU or the World Health Organisation, including the Joint Research Centre (JRC), European Environment Agency (EEA), and International Agency for Research on Cancer (IARC). These contacts have been useful in the exchange of knowledge, discussion on setting priorities in environment and health, understanding the interface with policy development at the EU-level, as well as awareness of the scientific impact on society and the social impact of non-action (e.g. EEA report: *Late Lessons from Early Warnings *[17]). So far a structured participation of representatives from the policy field has been rare, but there are a few exceptions. Policy makers participated as consortium members in an EU-funded project on Good Practice in exposure reduction options in the field of transport and health; and in the field of indoor environment and health [18]. National government policy makers contributed in the analysis of good practice, and inputs on analysis and feasibility of the implementation of measures was a useful contribution, which was widely disseminated across Europe. However, there were also some more negative aspects. One issue was the failure to use or promote innovative measures, and the presumed difficulty of ‘selling’ some examples at the political level blocked the implementation of certain measures. Furthermore, some conservatism in complying with the fixed set of rules and regulations of the political system prevailed, and there was a lack of organisational opportunity to act and to obtain internal financial support from the project consortium.

In addition, there is no discussion about the available knowledge or the quantity or quality of results from research in the field of health and environment. This is confirmed by the views of the policy experts interviewed, although the sharing of knowledge and transfer to actors in other disciplines or other fields of work is less common.

All of this may reflect the fact that there is some reluctance within the scientific community in participating in the science-policy interface, as it appears to be a focus for “stiff competition”. Dabelko stated: “An information glut is flooding everyone who can influence public policy. The competition for eyes and ears is stiffer than ever. And many academics who are reluctant to stray beyond the narrow bands of disciplinary journals take that competition as confirmation that we should let policymakers find us, not the other way around“ [19]. The application of some actions in HENVINET, such as the role playing session and the workshop with policy makers, opened the interaction between scientists and policy experts. It can be argued that the topic of the workshop, climate change and health, remains at the stage of scientific fact finding and thus might be more open for interaction.

However, time is also needed for building a trans-disciplinary network. At the start of HENVINET scientists did not see the need to provide policymakers with information. The attitude was passive. The project leaders had to shift this attitude towards a more active one by finding the right activities and structure to enhance cooperation between disciplines. The results of the different actions demonstrate that the various exercises and presentations during the project to encourage network building have altered this passive stance of the various health and environment experts. For example, placing the scientist in the role of industry or local authority radically changed the position of some participants in the role playing session. Dabelko explains this situation well, “But if scientists don’t engage in policy discussions and make our work more widely available, then we lose the ability to complain about policy decisions. And we miss genuine opportunities to share our insights. And a range of so-called “experts,” whether from industry or advocacy, will engage whether we do or not. Scientists (…) need to be part of these policy debates. Otherwise we cede the ground, I think, needlessly” [19].

### Interviews, role playing session, voting system and stakeholder workshop

This paper has discussed a number of activities to stimulate and facilitate the interaction between policy makers, scientists and other actors from civil society and industry. It has been concluded that the institutionalisation of this interaction is not easy. A first threshold is the fact that within this project consortium members were present who were very sceptical about cooperation across disciplines. Most likely this will be a starting point for other networks as well. Different activities were undertaken to increase awareness among the scientific partners within the consortium. The role playing game proved to be a successful action.

The role play session illustrated the usefulness of stakeholder involvement in procedures that aim to provide policy advice based in scientific expertise. The social complexity of environment and health issues was clearly illustrated during the role play, indicating the added value for policy makers to be informed not only about scientific aspects of environment and health issues, but also about social aspects from a diversity of actor perspectives. The role play moreover was able to convince most of the participating experts of the usefulness of stakeholder involvement. One of the more sceptical experts in the end became one of the main defenders, and as a spokesman for the group vigorously presented the benefits both of the role play and stakeholder involvement to the non participating experts from HENVINET. Moreover some participating experts indicated that the use of a method like the role play would have been beneficial to their perception of their involvement in the HENVINET project development, as it gave them the opportunity to better express their opinion in an interactive and cooperative manner.

The voting session provided ideas about ten different issues. Such a session could be applied as a tool to illustrate different opinions, points of interest and linkages between stakeholders from different fields of expertise. The formulation of questions or statements has to be carefully considered. Discussion regarding the votes proved to be an easy way to collect additional arguments around the questions.

The application of a PRS was received very positively by the participants. Quick feedback on the questions presented and the subsequent discussion was considered useful. The system can be used to bring the opinions of a trans-disciplinary network to the table in a rapid and participatory way, and the different disciplines can contribute without any feeling of being in the minority.

The stakeholder workshop was used to bring together the scientific community and the policy community. The sharing of information about the knowledge required, and also about success in the implementation of policy measures, stimulated desire among the participants of the workshop for further contact. From the acknowledgement of shared problems a small ad hoc network was formed. The role of intermediary was undertaken by HENVINET. Such a role should be defined to build a bridge between scientific and policy communities in a trans-disciplinary stakeholder workshop. These actions as well as the network portal, the role of intermediary institutes, organisations or group of individuals should be further investigated. It was believed by experts interviewed, and also mentioned during the PRS session, that this role is crucial for the survival of a trans-disciplinary network. This role could be compared to what Jeffrey calls the intermediary role [8].

In any stakeholder workshop one has to consider that there are some limitations to its success. Most of these limitations have to do with lack of communication between the expert, the non-expert stakeholders and the policy regulators. A human obstacle is that some people do not change their minds, which may be a failure attributed to the project actors. The lessons learnt from risk communication are that there has to be trust in the intermediary, besides the quality of the scientific knowledge used. A regulatory obstacle may be that local politics can conflict with national/international regulations. A practical reason can be financial: the resources are needed right now in order to achieve the goals, but are not available; and politically: it may be more acceptable to invest in the domains where there are the most visible problems, while it might be less effective to solve these. These same arguments are also true for the success or failure of the network portal.

### Networking portal

The action considered being more influential and most durable of those applied in HENVINET was the creation of a networking portal. The networking portal has the potential to be an effective tool to facilitate the sharing of knowledge and communication between stakeholders. The drawback of the networking portal is that each contributing part in such a network waits for the initiative of another actor (for example to supply content), and that success depends on the actions of a few leading stakeholders in the network. Furthermore, there are many other hurdles related to these types of networks: differences in the basic knowledge of actors, the different perceptions and perspectives of policy relevance, gaps in communication or communication language, dependency on funds, and the uncertainty of scientific knowledge. The benefits of a network of trans-disciplinary nature include: building of alliances with the private sector and civil society; building of new ways of communicating messages for the public. Stern et al. state that “the participation of both scientists and non-scientists is necessary for careful consideration of the implications of decision rules” [20,21] and therefore in contributing to the formation of policy measures.

The network portal provides a supportive structure for inter- and transdisciplinary cooperation, but such a platform needs continuous participation in order for it to become an active network. It has been proposed that skilled intermediaries are useful players to help policy makers engage with the research community. The example of the professionals in organisations like EEA, IARC and JRC are the given as intermediaries at the European level. At a more national, regional or urban levels these kinds of intermediaries are less available or even absent. While stakeholders from the policy field indicated in interviews that a trans-disciplinary network on health and environment would be a useful addition in this domain, they did not give clear answers on how to fill the role of intermediaries who could interact between the science and policy domains.

Social networking portals, role-play, stakeholder workshops or a Personal Response System applications are means to bring the different stakeholders together. They were applied here partly to get input on the required structure of the proposed network.

The HENVINET experience demonstrated that the networking portal is a tool suitable for disseminating knowledge, but it will never be the sole source for information- rather a complementary tool for policy makers. The networking function enables stakeholders across disciplines and domains to find the experts, but it does not provide policy makers with the insight to engage with the research community in a way which connects with scientific thinking. Therefore it can be concluded that the role of intermediaries is in essence not replaced by the portal.

## Conclusion

The answers to the research question concerning the needs of an inter- or trans-disciplinary network was provided by the interviews. The role of a network in dissemination was identified as an interacting role with different actors, but even more specifically to secure a closer collaboration between policy makers and researchers. This role is clearly specified in the responses of the interviewees and confirms the need for an integrated approach and the formation of a network.

HENVINET developed a social networking portal to enable stakeholders across disciplines and domains to find the experts, but it did not provide policy makers with the insight to engage with the research community in a way which connects with scientific thinking. Therefore it can be concluded that the role of intermediaries is in essence not replaced by the portal. The other applied tools provided insight in other domain’s thinking and acting, but do not have a role in the positioning of a network.

Several methods to form an inter- or trans-disciplinary network were applied. These methods, a role playing session, a personal response system and a stakeholder workshop were successful in increasing awareness among scientific partners about their role towards the policy domain. As supporting activity these methods can be used in building new networks. None of these methods can be used as the sole method to form a network.

The concept of the integration of science and policy within the environment and health fields using social networking principles has been outlined. This endeavour was envisioned at the beginning to be a purely scientific quest. It was anticipated that with the right group of professionals within the project consortium, the bridge to policy experts with regard to policy priorities for example, would naturally follow. In building bridges towards policy interpretation though, the limitations of a purely scientific undertaking were clearly demonstrated. Due to the many uncertainties and limited specialized knowledge, no scientist or group of scientists stepped outside their own niche and dared to use their knowledge to initiate discussions or to answer difficult questions about policy relevance. Similar conclusions have been drawn from other projects. One example of this is provided by a project involving a working group of scientists, governmental experts and policy representatives, mostly involved in the work of the Flemish Centre for Environment and Health, where they prepared an action-plan for the interpretation and use of policy for human biomonitoring data [14,22].

Participatory and dialogue based processes are available to combine scientific or practical expertise with policy and decision making. The main benefit of the different actions undertaken by HENVINET was to bring together people from different disciplines and domains. The participation by different actors in the actions brought scientists and policy experts closer together. The combination of actions was productive at the moment of performance. It is not clear what the longer lasting effects of these actions will secure. The social networking portal is a transparent tool with a lot of potential, but the role of the scientists in a social portal is not yet clear. This lack of clarity is a major threshold for scientists to overcome in order to fully participate. The policy expert is open to interdisciplinary activities, seeks transparency in problem identification based in integrative problem description and wants a clear knowledge transfer. These are essential ingredients for building a constructive and sustainable network of multiple stakeholders. The different actions provided by HENVINET contributed to these ingredients, but only for a short period. The social networking portal aimed to contribute to sustainable and continuing network building. It is yet too early to conclude if the social portal will be successful in contributing to building a trans-disciplinary network in the field of environment and health.

## Competing interests

The authors declare that they have no competing interests.

## Authors' contributions

HENVINET project: AB managed the project and contributed with analysis, discussion, and expertise to the networking activities presented.

Interviews: PvdH organized the setup of the interviews.

Social Networking Portal: SR and AY managed the portal.

Role-Play Scenario: PvdH and HK developed and moderated the role-play scenario.

Voting Session: PvdH organized the voting session.

Stakeholder Workshop: HK and DL organized the workshop. PvdH and HK led the workshop, and DL contributed to the workshop. SR recorded and summarized the workshop.

All authors revised the article and approved the final version.

## Supplementary Material

Additional file 1 **Protocol for HENVINET stakeholder consultation** Description of data: Protocol to carry out interviews with stakeholders for stakeholder consultation on their needs and concerns directed at the support of policy making in the field of environment and health and at the use of Decision Support Tools (DST) in support of policy making. The protocol was created to ensure that the interviews are documented in such a way that the results be synthesised into an overview report. Click here for file
