# Supplementary material for: The challenge of social networking in the field of environment and health
Source: Environ Health. 2012 Jun 28;11(Suppl 1):S15. doi: 10.1186/1476-069X-11-S1-S15 (PMC3388437; doi:10.1186/1476-069X-11-S1-S15)
Supplement: Additional file 1 — Protocol for HENVINET stakeholder consultation Description of data: Protocol to carry out interviews with stakeholders for stakeholder consultation on their needs and concerns directed at the support of policy making in the field of environment and health and at the use of Decision Support Tools (DST) in support of policy making. The protocol was created to ensure that the interviews are documented in such a way that the results be synthesised into an overview report. [file 1476-069X-11-S1-S15-S1.pdf]

Protocol for HENVINET stakeholder consultation

A. INTRODUCTION TO THE INTERVIEWER

This protocol aims:

- to facilitate the interviews with stakeholders[1] on their needs and concerns that may be relevant to HENVINET; these concerns and needs are directed at the support of policy making in the field of environment and health and at the use of Decision Support Tools (DST) in support of policy making.
- to ensure that the results of the interviews are documented in such a way that the results of all interviews can be synthesised into an overview report.

The interview has four steps:

1. Introduction
2. Explanation of Environment and Health;
3. Explanation of HENVINET
4. Comments, suggestions, concerns relevant to HENVINET
5. Other questions

The comments and answers should be documented in the template that is provided at the end of this protocol.

[1] The term "stakeholder" encompasses all groups potentially interested in HENVINET results, including policy makers and research networks.

B. GUIDANCE FOR THE INTERVIEW

The interview consists of five parts.

Part I: Introduction to the interview (purpose, structure)

The interviewer should explain the purpose and structure of the interview. The purpose of the interview is to draw up an overview of the concerns and needs that potential users of HENVINET results may have in the field of E&H. This will be used to better focus HENVINET on the specific needs of stakeholders.

The views expressed by the interviewee will be regarded as a personal opinion, not as an official view of the organisation that the interviewee is affiliated to.

- The interviewer should ask details of the interviewee (see template in Section C).

Part II: Explanation of Environment & Health

The interviewer should explain E&H and the role of risk assessment in E&H.

- **What is Environment & Health?**
  Although much progress has been made in improving the quality of air, water and soil, the situation remains far from satisfactory from a health point of view. The EU therefore strives towards closer cooperation between the health, environment and research areas.

  The European Commission adopted in 2003 an EU Strategy on Environment and Health, with the overall aim to reduce diseases caused by environmental factors in Europe. This was followed up by the European Environment and Health Action Plan 2004-2010 which proposes an Integrated Information System on Environment and Health to render the assessment of the environmental impact on human health more efficient.

  Today, there is evidence that factors such as particulate matter in the air, noise and ground-level ozone damage the health of thousands of people every year. Environmental pollutants, including pesticides, endocrine disruptors, dioxins and PCBs persist in the environment, accumulating over time and we do not know enough about their long-term effect on our health. The effect of the environment on health is thus a major concern of the European public: in a recent survey, some 89% are worried about the potential impact of the environment on their health. Furthermore, new technologies, changing lifestyles, work and life patterns, present new and sometimes unexpected impacts on the environment and its influence on health.

- General comments on the needs and concerns related to the broad E&H issue should be invited. These should be summarised in the Template.

Part III: Explanation of HENVINET

The interviewer should explain HENVINET and what it hopes to achieve.

What is HENVINET?
HENVINET is a multidisciplinary network with 30 participating institutes to support E&H. It has been established as a project under the sixth Framework Programme for Research, running from 2006-2010. It aims to contribute to E&H in a number of ways to support such informed policy making:

- *support policy making directly, by organising interaction between the scientific community and policy makers and by providing guidance and more reliable tools;*

- *support policy making indirectly by contributing to an integrated environment and health information system, thereby improving the knowledge on causal links between environmental factors and human health;*

- *validate tools and results with emphasis on the four EHAP priority health diseases, and provide structured information overview that may be utilised by other actors relevant to Environment and Health Strategy;*

- *review, exploit and disseminate knowledge on environmental health issues based on research and practices, for wider use by relevant stakeholders; it will give particular attention to four priority diseases (asthma and allergies; cancer; neurodevelopmental disorders; endocrine disruptors)*

- *establish an overview of results, activities, projects and tools existing in Europe and promote stakeholder networking through workshops and project meetings;*

- *evaluate Decision Support Systems relevant to integrated management of environmental health risks.*

Overall the main objective of HENVINET is to establish a long-term co-operation between researchers, policy makers and other stakeholders in the area of environment and health research and assessment.

Part IV: Comments, suggestions, concerns relevant to HENVINET

- ➢ The interviewer should proceed with the questions of Part IV of the template on the personal experience of the interviewee and the needs of the stakeholders related to E&H.

Part VI: Other questions

- ➢ The interviewer should discuss possibilities for maintaining contact with HENVINET, ask about possible other useful contacts and invite any concluding comments (see Part V of the template).

C. TEMPLATE: QUESTIONS AND ANSWERS

Interview of <name>

Interviewer: <name>
Date of interview: <date>

Part I. Details of interviewee

Question 1. ***Name etc***	
Name	
Affiliation	
Address	
Country	
Telephone	
Email	
Function	

Question 2. What ***type of stakeholder*** are you (tick one or more)

***Type of use** of results of HENVINET*	***Tick***
Developing policy/legislation related to E&H	
Applying policy/legislation related to E&H	
Addressing stakeholder interests (Industry, NGO, …)	
Providing public information on E&H	
Medical practice	
Consulting	
Developing risk assessment / decision support	
Research	
Other (please specify):	
…	

Part II. General comments on Environment & Health

Question 3. ***Broad comments*** on priorities, needs and concerns related to E&H
…

Part III. Explanation on HENVINET

[No questions]

Part IV. Comments, suggestions, concerns about HENVINET

Question 4. Do you have comments, suggestions, and concerns about the **network HENVINET**? What do you expect from HENVINET?
…

Question 5. Do you have comments, suggestions, and concerns about the

following **specific objectives** of HENVINET?

HENVINET intends to identify **the most effective preventive policy measures**. Assuming the acceptance of all major stakeholders, and assuming the existence of a (co-) causal link what do you think would be the potentially most effective preventive policy measures to reduce the risk of environmental pollution which will lead to asthma and allergies/cancer/neurodevelopmental disorders/ endocrine disruptor mediated diseases?
…

HENVINET intends to evaluate existing **standards in the light of concerns of vulnerable groups**. Do you have comments, suggestions, and concerns?
…

HENVINET intends to evaluate **Decision Support Tools**. Do you have suggestions for:<br>(a) issues for which DSTs are particularly useful?<br>(b) particular aspects to be addressed in the evaluation?<br>(c) are you aware of any software tool(s), assessment protocol)s) or analytical framework(s) that could be used to analyse the environment and health related information?
…
HENVINET aims to provide synthesised **information to society**. What are important **issues to address**?
…

Do you have suggestions for **effective dissemination** of HENVINET results to **media and professionals**?
…

Do you have suggestions for feeding **information** on health effects into the **policy making arena**?
…

HENVINET intends to identify **emerging issues**. Do you have suggestions on how to identify these? And do you see particular emerging issues that HENVINET should give attention to?
…

HENVINET intends to identify **priority areas for research.**<br>(a) What are important gaps of knowledge?<br>(b) Do you have suggestions for how to prioritise research?
…

Where do you see the main **obstacles for action** at the EU level to address E&H issues?
…

Question 6. Henvinet will address four **priority diseases**:

- Asthma and Allergies
- Cancer
- Neurodevelopmental Disorders
- Endocrine disrupter-mediated diseases

For each disease, HENVINET intends to review the **state of knowlegde**. Do you have particular suggestions on what not to overlook within these fields?
Asthma and allergies: …
Cancer: …
Neurodevelopmental Disorders: …
Endocrine disrupter-mediated diseases: …
For all diseases: …

For each disease, HENVINET intends to recommend **good practice**. Do you have particular suggestions on what to address within these fields?
Asthma and allergies: …
Cancer: …
Neurodevelopmental Disorders: …
Endocrine disrupter-mediated diseases: …
For all diseases: …

For each disease, HENVINET intends to address **Frequently Asked Questions**. Do you have particular suggestions on what to address here?
Asthma and allergies: …
Cancer: …
Neurodevelopmental Disorders: …
Endocrine disrupter-mediated diseases: …
For all diseases: …

Question 7. Do you see **other important issues** that HENVINET could consider to address?
…

Part IV. Future development

Question 8. Are you interested in ***maintaining contact*** with HENVINET?

Mode of contact	***Tick***
No further contact	
On HENVINET's Stakeholder Emailing List	
Available for any next stakeholder consultation	
Invited to workshops and meetings	
Available for evaluation of quality of our knowledge on variety of aspects of the causal chain between environmental stressors and human health? *	
Contribute to HENVINET's work to be done. If yes, please specify any preference:	
…	

Question 9. Would you like to suggest ***other colleagues or contacts*** that you think would be interested in being involved in HENVINET?

Name	***Organisation***	***Contact details***
Add rows when needed		

Question 10. ***Any other comments***
…

* Explanation to question: One of the goals of Henvinet is to assess the quality of the knowledge available in order to identify gaps and weak points. Under situations of high uncertainty and complexity, different stakeholders will have different points of view regarding the quality of the knowledge available. Would you be willing to participate in a second interview where you would be asked to perform an evaluation of the quality of our knowledge on a variety of aspects of the causal chain between environmental stressors and human health?
